# miR-181a Regulates Inflammation Responses in Monocytes and Macrophages

**DOI:** 10.1371/journal.pone.0058639

**Published:** 2013-03-13

**Authors:** Weidong Xie, Mengnan Li, Naihan Xu, Qing Lv, Nunu Huang, Jie He, Yaou Zhang

**Affiliations:** Shenzhen Key Lab of Health Science and Technology, Division of Life Sciences & Health, Graduate School at Shenzhen, Tsinghua University, Shenzhen, China; University of Colorado Denver, United States of America

## Abstract

miR-181a has been presumed to target the 3′-untranslated regions (3′-UTR) of IL1a based on software predictions. miR-181a and IL1a have opposite expression levels in monocytes and macrophages in the inflammatory state. This led us to suspect that mir-181a has an important function in regulating inflammatory response by targeting IL1a. Fluorescence reporter assays showed that miR-181a effectively binds to the 3′-UTR of IL1a. The anti-inflammatory functions of miR-181a were investigated in lipopolysaccharides (LPS)-induced Raw264.7 and phorbol 12-myristate 13-acetate (PMA)/LPS-induced THP-1 cells. We found that miR-181a mimics significantly lowered IL1a expression levels in these cells and, interestingly, miR-181a inhibitors reversed this decrease. In addition, miR-181a mimics significantly inhibited increase in the levels of inflammatory factors (IL1b, IL6, and TNFa) in these cells. Furthermore, miR-181a mimics and inhibitors decreased and increased, respectively, production of reactive oxygen species in PMA/LPS-induced THP-1 cells. These results indicate that miR-181a regulates inflammatory responses by directly targeting the 3′-UTR of IL1a and down-regulating IL1a levels. Interestingly, we found that miR-181a inhibited production of inflammatory factors even in IL1a-induced THP-1 cells, suggesting that the anti-inflammatory effects of miR-181a possibly involves other targets in addition to IL1a. Thus, we provide the first evidence for anti-inflammatory effects of miR-181a mediated at least in part by down-regulating IL1a.

## Introduction

Inflammation is a complex biological, physiological, and pathological response of vascular tissues to harmful stimuli, such as infection and tissue damages [1]. Inflammation is a “double-edged sword”. Without inflammation, wounds and infections cannot heal. However, inflammation also causes tissue damages and host diseases. Many diseases such as diabetes [2], obesity [3], cardiovascular diseases [4], and cancer [5] are associated with inflammation responses. Inflammation factors are mainly secreted from monocytes and macrophages. However, the exact mechanism of regulation of these inflammatory factors by physiological and pathological stimuli remains unclear.

miRNAs are short ribonucleic acid molecules usually composed of 20 to 25 nucleotides [6] that can regulate mRNA transcription and protein translation by targeting the 3′-untranslated regions (3′-UTR) of target genes [7]. miRNAs are involved in various physiological responses and pathological processes. Increasing evidence suggest that miRNAs are involved in inflammatory responses [8]. miR-146a regulates TNF-α-induced IL-8 production in mesenchymal stem cells and differentiated lung epithelial-like cells [9], [10]. miR-378-3p is essential in limiting macrophage expansion and alternative activation during type 2 inflammatory settings by targeting the PI3K/Akt-signaling pathway [11]. miR-155 promotes inflammation by targeting SHIP1 [12]. miR-21 negatively regulates programmed cell death 4, promotes NF-κB activation, and suppresses IL-10 [13]. miR-29 exacerbates inflammation by targeting the negative regulators of NF-κB signaling [14]. MicroRNA-181b regulates NF-κB-mediated vascular inflammation by targeting importin-α3 [15]. However, the mechanisms by which these miRNAs promote and regulate inflammation are not completely understood.

miR-181a is mainly involved in the regulation of tumor growth and the immune system [16]. In our preliminary study using the software programs Find Tar3 (http://bio.sz.tsinghua.edu.cn), TargetScan (http://www.targetscan.org/), and miRBD (http://MiRBD (http://mirdb.org/miRDB/), miR-181a was predicted to have several target sites of inflammatory factors. In mice, miR-181a has been predicted to directly target the 3′-UTRs of several key inflammatory or signaling pathway factors such as IL1a, MAPK1, TNFa, and IL6, among others. In humans, miR-181a has been predicted to target the 3′-UTRs of IL1a, MAPK1, TNFa, and TLR4. Therefore, miR-181a may exert its anti-inflammatory effects by simultaneously targeting. Among these sites, the 3′-UTR of IL1a where miR-181a is predicted to bind is relatively conserved in several animal species including mice and humans. In this study, we investigated whether miR-181a regulates inflammatory responses by targeting IL1a in lipopolysaccharides (LPS) or phorbol 12-myristate 13-acetate (PMA)/LPS-induced Raw264.7 and THP-1 cells.

## Methods

### Cell Culture and Induction of Inflammation

#### Raw264.7 cells

Raw264.7 cells were obtained from the Cell Resource Center of the Shanghai Institute for Biological Sciences, Chinese Academy of Sciences, China. The cells were cultured in DMEM (high glucose, Invitrogen) supplemented with 10% FBS and incubated in a humidified atmosphere of 5% CO_2_ at 37°C. The cells were seeded into six-well plates at a density of 5×10^5^ cells per well. Inflammation was induced at a final concentration of 2 µg/ml LPS (Lot. No. L4391, Sigma-Aldrich, USA) after 12 h of attachment. Cell and media samples were collected at 0, 6, 12, and 24 h after LPS induction. Briefly, cells were washed with PBS twice after the media samples were collected. A total of 200 µl of cell lysis buffer (50 mM Tris–HCl, 4 M urea and 1% Triton X-100, pH 8.0) was then added into the wells for cell sample collections. All samples were immediately stored at −80°C for future biochemical assays.

#### THP-1 cells

THP-1 cells were obtained from KeyGEN Biotech, Nanjing, China. This cell line was grown in suspension cultures for passages. The cell culture conditions were similar to those of Raw264.7 cells. The cells were seeded into six-well plates at a density of 5×10^5^ cells per well, with simultaneous addition of PMA (Lot. No. 79346, Sigma-Aldrich, USA) at a final concentration of 200 ng/ml. Most of the suspended THP-1 cells attached to the plate bottom differentiated from monocytes to macrophages after 12 h of PMA stimulation. The cell medium was replaced with fresh medium (DMEM+10% FBS, without PMA), and inflammation was stimulated simultaneously by adding LPS at a final concentration of 2 µg/ml (Sigma, USA) to the medium. Methods of cell and medium sample collection and preservation were identical to those of Raw264.7 cells. In this study, PMA was used to induce the formation of macrophages in THP-1. LPS was used to further induce inflammatory responses. PMA combined with LPS can produce more inflammatory factors than PMA or LPS alone.

### miRNA or siRNA Transfection

miRNA mimics were synthesized by Shanghai GenePharma Co. (Shanghai, China) (Table 1). Human pre-miR™ miRNA precursor anti-miR™ miRNA inhibitors (has-mir-181a and negative controls) were purchased from Ambion® (Life Technologies, USA). miR-181a precursors and its inhibitor were designed according to the miRBase sequence database (http://microrna.sanger.ac.uk). siRNA duplexes with random sequence were used as negative control. Raw264.7 and THP-1 cells were seeded into six-well plates for 12 h. THP-1 cells were simultaneously stimulated with PMA at a final concentration of 200 ng/ml after seeding into the culture plate. The cells were replaced with fresh medium (DMEM+10% FBS) and transfected with siRNA or miRNA mimics at a concentration of 50 pmol/well to 60 pmol/well using Lipofectaime 2000 (Invitrogen™, USA) according to the manufacturer’s instructions. After 6 h to 12 h of transfection, the medium was replaced with fresh medium containing 2 µg/ml LPS. The cell medium and lysis samples were then collected according to the aforementioned protocol after 24 h of LPS induction. In another trial, THP-1 cells were cultured with exogenous IL1a (at a final concentration of 0.1 ng/ml) instead of LPS to test whether miR-18a mimics affect IL1a-induced inflammation.

**Table 1:** Nucleic acid sequences for miRNAs and siRNA mimics.

Gene names	Sequences (5′ to 3′)
miR-181a mimics	AACAUUCAACGCUGUCGGUGAGU
Negative controls	UUCUCCGAACGUGUCACGUTT
miR-181a inhibitors	ACUCACCGACAGCGUUGAAUGUU
miRNA inhibitor NC	CAGUACUUUUGUGUAGUACAA
siRNA NC	UUGUACUACACA AAAGUACUG
siIL1a poor for mouse(Raw264.7 cells)	IL1a siRNA (m) sc-39614)(Santa Cruz Biotechnology, Inc.)
siIL1a poor for human(THP-1 cells)	Si-1: ACUUGUUUAUUGCCACAAA
	Si-2: GUAUAAUUCGAGCCA AUGA

### MicroRNA Expression System

The pre-miRNA (mir-181a precursor, 60 nt to 70 nt) and flanking 300 nt sequence were cloned into a pCMV-MIR vector (OriGene, USA) and an empty pCMV-MIR was used as negative control. A stable transfection was conducted in Raw264.7 cells. Cell lines with stable expression of mir-181a precursors and mock controls were then selected using G418 (Sangon, Shanghai, China) at a final concentration of 1000 µg/ml. In THP-1 cells, a transient transfection was performed with 500 ng/well of plasmids in six-well plates. Other steps were conducted following the same protocol as that of miRNA transfection described above.

### miRNA q-PCR

miR-181a was analyzed using the TaqMan microRNA assay kit (Applied Biosystems, USA). The U6 gene was used as an internal control for normalization. Reverse transcription (RT) was conducted using a DNA Engine® Peltier Thermal Cycler (Bio-Rad, USA) with a thermocycler program consisting of 16°C for 30 min, 42°C for 30 min, 85°C for 5 min, and a hold step at 4°C. All Q-PCR measurements were performed using a 7300 Real Time PCR system (Applied Biosystems, USA) with a thermocycler profile consisting of 95°C for 10 min, followed by 40 cycles of 95°C for 15 s and 60°C for 60 s according to the manufacturer’s protocol. Fold change was calculated using the 2^−ddCt^ method of relative quantification. All experiments were conducted in triplicates.

### mRNA q-PCR

Total RNA was extracted using the TRIZOL reagent (Invitrogen) according to the instructions of the manufacturer. RT was performed using a PrimeScript™ 1st Strand cDNA Synthesis Kit (Takara, Dalian, China), and cDNA fragments were amplified using Taq DNA Polymerase (Takara, Dalian, China) according to the instructions of the manufacturer. In this study, these genes were chosen for quantitative real-time PCR (q-PCR), and primers were synthesized from Invitrogen (Table 2). Glyceraldehyde-3-phosphate dehydrogenase was used as an internal control for normalization. RT was conducted using an Alpha™ Unit Block Assembly for DNA Engine® systems (Bio-Rad, USA) with a thermocycler program consisting of 42°C for 30 min, 85°C for 5 min, and a hold step at 4°C. Q-PCR analysis was conducted using SYBR® Green I dye following the manufacturer’s protocol (Code No. DRR820A, Takara, Dalian, China) in an ABI PRISM 7300 Real time PCR System (Applied Biosystems, USA). Q-PCR analysis was performed in two steps: (1) cDNA samples were pre-denatured at 95°C for 30 s at the first stage, (2) denatured cDNA samples were amplified with 40 cycles at 95°C for 15 s and at 60°C for 31 s at the second stage. Data were analyzed by the raw relative quantitation method (2^−ddCt^).

**Table 2:** Primers for energy metabolism genes.

Gene names	NCBI Accession No.	Primers (5′ to 3′)	Sizes(bp)
Mouse IL1a	NM_010554.4	Forward: TCTGCCATTGACCATCTC	182
		Reverse: ATCTTCCCGTTGCTTGAC	
Mouse IL1b	NM_008361.3	Forward: GTTCCCATTAGACAACTGC	199
		Reverse: GATTCTTTCCTTTGAGGC	
Human IL1a	NM_000575	Forward: ATCATGTAAGCTATGGCCCACT	131
		Reverse: CTTCCCGTTGGTTGCTACTAC	
Human IL1b	NM_000576	Forward: CTCGCCAGTGAAATGATGGCT	144
		Reverse: GTCGGAGATTCGTAGCTGGAT	
Mouse GAPDH	NM_008084	Forward: TCTCCTGCGACTTCAACA	178
		Reverse: TGGTCCAGGGTTTCTTACT	
Human GAPDH	NM_001256799	Forward: GGAGCGAGATCCCTCCAAAAT	197
		Reverse: GCTGTTGTCATACTTCTCATGG	

### Western Blot Analysis

Cell extracts were separated by SDS-PAGE (12%) and transferred to PVDF membranes. The membranes were blocked with 5% non-fat dry milk for 1 h and left to react overnight with goat polyclonal anti-IL1a (Santa Cruz, Japan) and mouse polyclonal β-actin antibody with dilutions of 1∶1000 in both cases. After rinsing, the membranes were soaked in blocking buffer with anti-rabbit secondary antibody (1∶1000) for an hour. After rinsing again, the membranes were used to visualize the blots by the method of chemiluminescence (KPL, USA).

### ELISA

Mouse IL1a, IL1b, IL6, and TNFa and human IL1b, IL6, and TNFa were assayed using the QuickEIA™ ELISA method (DAKEWE Biotech Co., Ltd., Beijing, China). Human IL1a was measured by the ABC-ELISA method (Shanghai Westang Bio-tech, China). Total protein concentration was determined at 595 nm using the Bradford assay (Bio-Rad) on a spectrophotometer (TECAN, Switzerland). ELISA data of cell lysate samples were normalized with protein concentrations. Other steps were carried out as described in protocols accompanying the kits.

### Luciferase Reporter Assays

Luciferase assays were performed as described in the kit protocol (Promega, USA). Briefly, the 3′-UTRs of mouse IL1a (nt 10 to 286) and their corresponding mutated 3′-UTRs were amplified by PCR using the primers shown in Table 3. These target sequences were cloned into the pRL-TK reporter vectors (Promega, USA). Cos-7 cells were seeded into 24-well plates at a density of 1×10^5^. Co-transfection was performed the following day using 300 ng of constructed plasmid and 20 pmol of each miRNA using Lipofectamine 2000. Cell lysates were collected after 24 h of transfection. Renilla luciferase activities were measured using a Luciferase Reporter Assay System (Promega, USA). Each experiment was conducted in triplicate on a Thermo Scientific Varioskan Flash Spectral Scanning Multimode Reader (USA). Total protein concentration was determined at 595 nm using the Bradford assay (Bio-Rad) on a spectrophotometer (TECAN, Switzerland). Luciferase activity was normalized by the total protein content.

**Table 3:** Primers of mouse IL1a 3′-UTR and its mutated fragments.

Gene names	Primers (5′ to 3′)
IL1a-3′UTR	Forward: GCCTCTAGATATTTCGGGAGTCTATTC
	Reverse: GCCCTCGAGTAGAGTCTTTTTGATCCTC
IL1a-3′UTR-mut	Forward: CGTAAGTTTACCCTCTTTGTAAGAG
	Reverse: TTTAGAATTACAGAGACTCAGCACA

The cloned sequence:

TATTTCGGGAGTCTATTCACTTGGGAAGTGCTGACAGTCTGTATGTACCATGTACAGGAACCTTCCTCACCCTGAGTCACTTGCACAGCATGTGCTGAGTCTCTGTAATTCTAAATGAATGTTTACCCTCTTTGTAAGAGAAGAGCAAACCCTAGTGGAGCCACCCCGACATATGATACTATCTGTTATTTTAAAGAGTACCCTATAGTTTGCTCAGTACTAATCATTTTAATTACTATTCTGCATGGCATTCTTAGGAGGATCAAAAAGACTCTA.

The mutated sequence:

TATTTCGGGAGTCTATTCACTTGGGAAGTGCTGACAGTCTGTATGTACCATGTACAGGAACCTTCCTCACCCTGAGTCACTTGCACAGCATGTGCTGAGTCTCTGTAATTCTAAACGTAAGTTTACCCTCTTTGTAAGAGAAGAGCAAACCCTAGTGGAGCCACCCCGACATATGATACTATCTGTTATTTTAAAGAGTACCCTATAGTTTGCTCAGTACTAATCATTTTAATTACTATTCTGCATGGCATTCTTAGGAGGATCAAAAAGACTCTA.

### Reactive Oxygen Species (ROS) Assay

ROS were assayed using a kit provided by the Beyotime Institute of Biotechnology (Haimen, Jiangsu, China). Briefly, the fluorescence probe DCFH-DA was transported into the cells and hydrolyzed into DCFH. Since DCFH cannot be transported through the plasma membrane, it stays inside the cells. Intracellular active oxygen binds to DCFH and causes DCFH to emit fluorescence, which can be detected through fluorescence microscopy (excitation wavelength: 485 nm; emission wavelength 525 nm). In this study, THP-1 cells (5×10^4^ per well) were seeded into 24-well plates and incubated in media containing PMA (200 ng/ml final concentration) for 12 h. After replacing the media, the cells were transfected with miRNA or siRNA mimics for 12 h. LPS (2 µg/ml final concentration) was then added to the media for 6 h to 24 h. Subsequently, media from each plate was replaced with fresh media (without FBS) containing DCFH-DA (10 µM final concentration) and the plates were incubated for 20 min. The cells were then washed three times with fresh media (without FBS) and immediately observed under a fluorescence microscope (LEICA DMI6000B, German). PMA alone produced ROS, and LPS further increased ROS production. However, no significant effects were observed in ROS production using LPS alone.

### Statistical Analysis

Data are expressed as mean ± SD. Statistical significance of data was evaluated by conducting ANOVA. Newman–Keuls comparison was used to determine the source of significant difference where appropriate. *P*<0.05 was considered statistically significant.

## Results

### Binding Sites for miR-181a in the IL1a 3′-UTR

The following four programs predicted that miR-181a targets the mouse and human IL1a 3′-UTRs:

Targetscan (http://www.targetscan.org/)

Miranda (http://www.microrna.org/)

Pictar (http://pictar.mdc-berlin.de/)

miRDB (http://mirdb.org/miRDB/)

**Figure 1:**
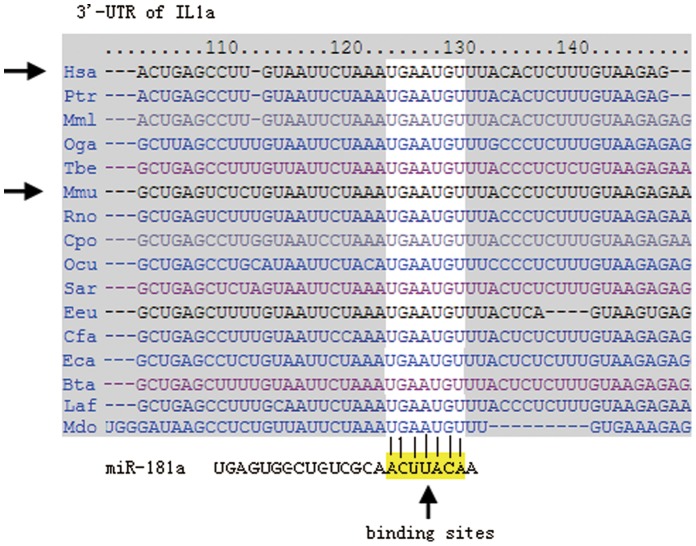
Presumed binding sites for miR-181a in the 3′-UTR of mouse and human IL1a. The binding sites are highly conserved.

Targetscan showed that the binding site for miR-181a in the IL1a 3′-UTR is relatively conserved (Figure 1). We next carried out experiments to confirm the binding of miR-181a in the IL1a 3′-UTR.

### Relationship of Endogenous miR-181a and IL1a Levels in Inflammatory Cells

**Figure 2:**
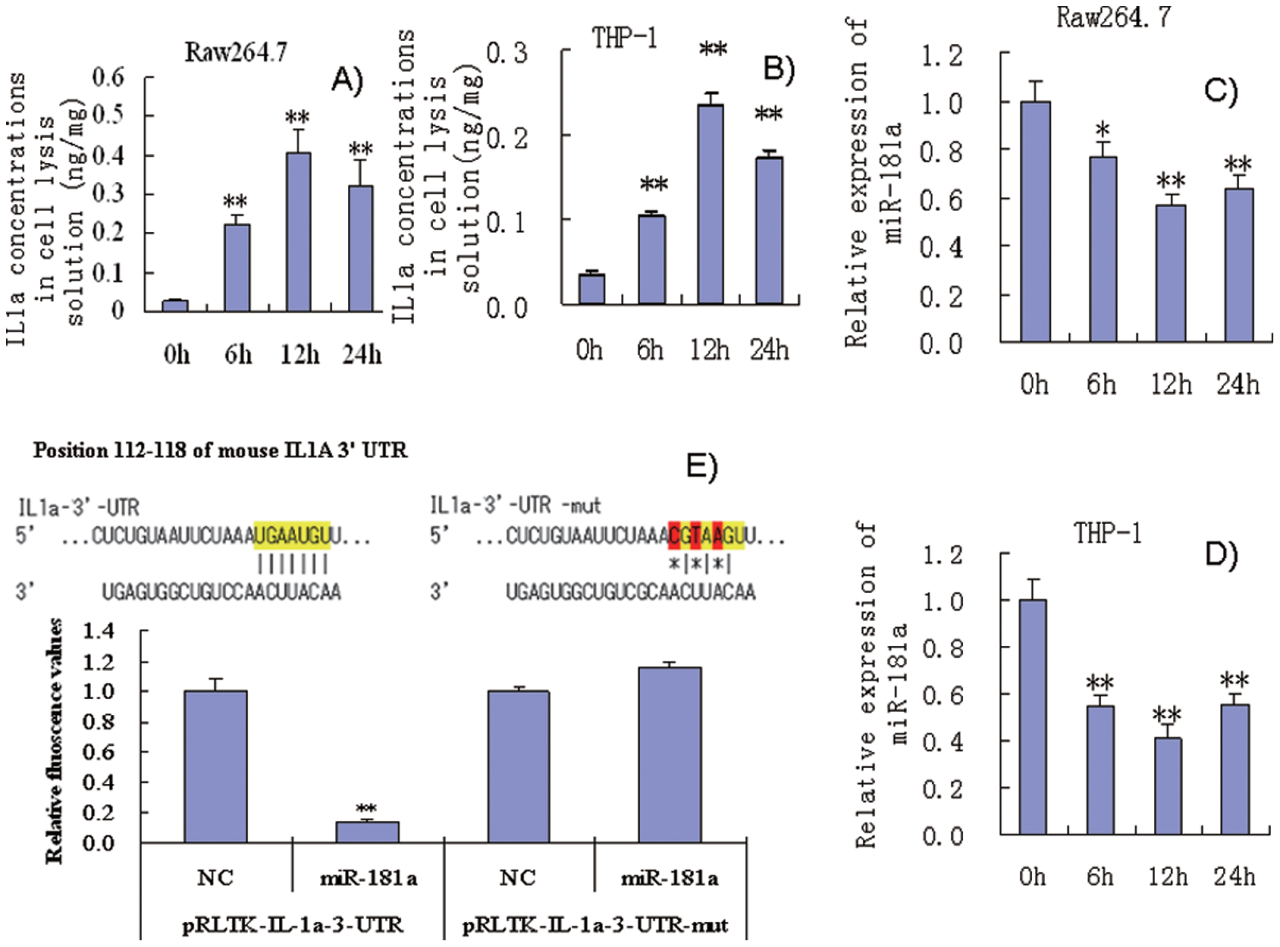
(A and B) miR-181a expression in LPS-induced Raw264.7 cells and PMA/LPS-induced THP-1 cells; (C and D) intracellular IL1a levels in LPS-induced Raw264.7 cells and PMA/LPS-induced THP-1 cells; (E) effects of miR-181a mimics on chemiluminescence values in Cos-7 cells (transfected with pRLTK containing L1a-3′-UTR or its mutated variant). Data are expressed as mean ± SD (n = 3), **P<0.01 vs. values at 0 h (A–D), **P<0.01 vs. negative control (NC, E).

Raw264.7 and THP-1 cells treated with 2 µg/ml of LPS showed significant increase in IL1a expression within 24 h (Figures 2A and 2B). However, IL1a concentration in the media was low and difficult to detect, unlike in cell lysis solutions. This suggests that IL1a primarily functions intracellularly. Interestingly, miR-181a levels were significantly decreased in these cells (Figures 2C and 2D). Thus, expression levels of miR-181a and IL1a were opposite in these cells, which is consistent with the prediction that miR-181a down-regulates IL1a by binding to its 3′-UTR.

### Examining Binding of miR-181a to the IL1a 3′-UTR by Luciferase Reporter Assay

Next, sequences from the miR-181a target site in the 3′-UTR of IL1a and their mutant variants were successfully cloned into pRL-TK vectors. The pRL-TK vectors contained 3′-UTR of IL1a and their mutant variants were transfected into Cos-7 cells, respectively. In luciferase reporter assays, transfection with miR-181a mimics significantly inhibited the chemiluminescence of Cos-7 cells transfected with pRL-TK-IL1a-3′-UTR compared with negative controls (NC) (Figure 2E). However, miR-181a mimics had no effect on the chemiluminescence of Cos-7 cells transfected with pRL-TK-IL1a-3′-UTR-mutated fragments in which the 3 bases in the miR-181a target sites were mutated. These results suggest that miR-181a specifically binds to the IL1a 3′-UTR. It is noteworthy that only the sequences that were found to be highly conserved in mice, humans, and other species from the IL1a 3′-UTR were cloned, and our results indicate that these sequences from the mouse IL1a 3′-UTR are sufficient for mirR-181a binding and regulation.

### Validation of Transfection Method on Inflammatory Cells

Most Raw264.7 and THP-1 cells emitted green fluorescence after 24 h of transfection with NC-Fam (a negative control with green fluorescence), confirming the efficacy of the transfection method we used (Figure S1, A and B). Furthermore, miRNA-181a levels were assayed after the cells were transfected with miR-181a mimics and its inhibitors. These cells showed extremely high levels of miR-181a even at 24 h of transfection with miR-181a mimics (Figure S1, C and D). However, miR-181a inhibitors significantly inhibited endogenous miR-181a expression 24 h after transfection. These results confirmed the suitability of our protocols for reliable functional assays of miR-181a.

### Effects of miR-181a Mimics on IL1a and IL1b mRNA Expression

**Figure 3:**
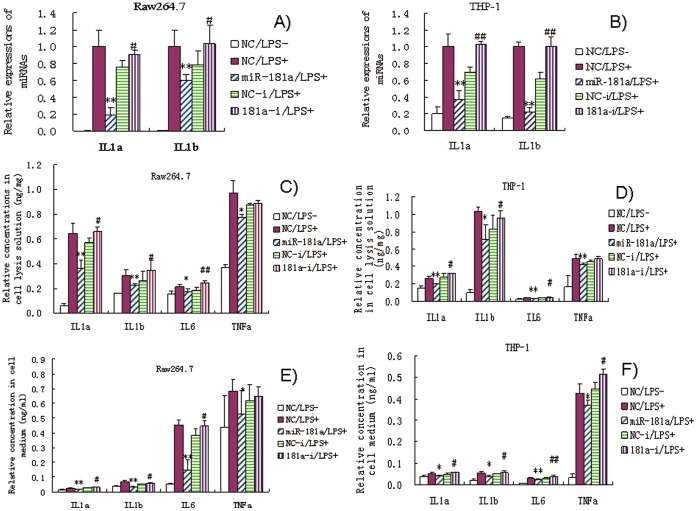
Effects of miR-181a mimics and inhibitors on IL1a and IL1b mRNA levels in LPS-induced Raw264.7 (A) and PMA/LPS-induced THP-1 cells (B); effects of miR-181a mimics and inhibitors on ILa, IL1b, IL6, and TNFa in cell lysates and media of LPS-induced Raw264.7 (C and E) and PMA/LPS-induced THP-1 cells (D and F) assayed by ELISA. Data are expressed as mean ± SD (n = 3), **P<0.01 vs. negative control (NC), ^#^ *P*<0.05, ^##^ *P*<0.01 vs. miRNA inhibitor negative control (NC-i). ‘NC-i’ is a single stranded nucleic acid used as negative control for miR-181a inhibitors (181a–i). ‘LPS + or −’, cells were treated with or without LPS in the case of Raw264.7 cells or PMA/LPS in the case of THP-1 cells.

After 24 h of LPS induction, levels of IL1a and IL1b mRNAs increased significantly in Raw264.7 and THP-1 cells (Figures 3A and 3B). However, miR-181 mimics significantly inhibited this increase compared with NCs. miR-181a inhibitors slightly increased IL1a and IL1b mRNA levels compared with their corresponding NCs. These results suggest that miR-181a represses IL1a and IL1b expression at the transcriptional level. We did not find a conserved site targeted by miR-181a in mice and human IL1b, so decided to focus on IL1a for further analysis.

### Effects of miR-181a Mimics on the Levels of IL1a, IL1b, IL6, and TNFa

IL1a, IL1b, IL6, and TNFa are secreted from Raw264.7 and THP-1 cells upon stimulation of inflammation. In order to quantitatively assay these responses, levels of IL1a, IL1b, IL6, and TNFa were assayed by ELISA in cell lysis solution and culture media. LPS significantly increased the levels of IL1a as well as IL1b, IL6, and TNFa in the cell lysis solution and cell culture medium (Figure 3C–3F). However, miR-181a mimics significantly inhibited this increase. In most cases, miR-181a inhibitors slightly increased the expression or secretion of IL1a, IL1b and IL6 proteins in either the cell lysis solution or culture medium. However, antagonism of miR-181a did not have any significant effect on TNFa levels in cell lysates. This suggests that, besides miR-181a, other miRNAs may also be involved in TNFa regulation. Interestingly, IL1a was found at low levels in cell culture media, suggesting that IL1a cannot be easily secreted from cells. Thus, IL1a may primarily act intracellularly as described above.

**Figure 4:**
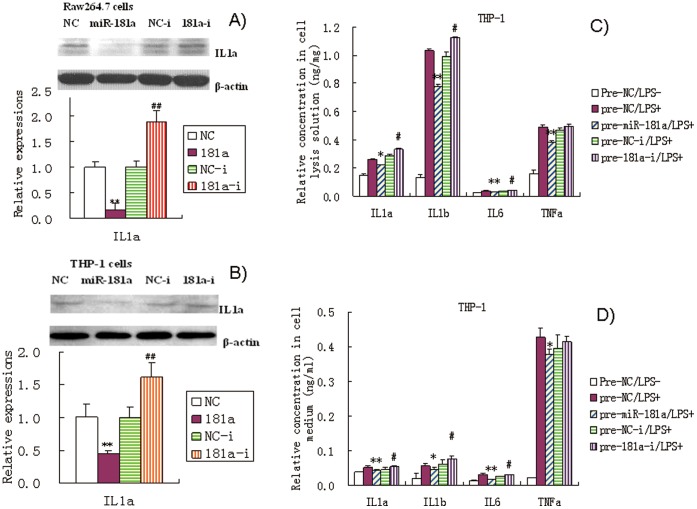
Effects of miR-181a mimics and inhibitors on ILa levels in cell lysis solution of LPS-induced Raw264.7 (A) and PMA/LPS-induced THP-1 cells (B) assayed by Western blot. Data are expressed as mean ± SD (n = 3–5), **P<0.01 vs. negative control (NC), ^##^ *P*<0.01 vs miRNA inhibitor negative control (NC-i). ‘NC-i’ is a single-stranded nucleic acid used as negative control for miR-181a inhibitors (181a–i). Effects of miR-181a precursors (pre-miR-181a) and its inhibitors on ILa, IL1b, IL6, and TNFa in cell lysates and culture media of PMA/LPS-induced THP-1 cells (C and D) assayed by ELISA. Data are expressed as mean ± SD (n = 3), *P<0.05, **P<0.01 vs. negative control (pre-NC), ^#^ *P*<0.05 vs. miRNA inhibitor negative control (pre-NC-i). ‘pre-NC-i’ is a single-stranded nucleic acid used as negative control for miR-181a inhibitors (pre-miR-181a–i). ‘LPS + or −’, cells were treated with or without LPS in the case of Raw264.7 cells or PMA/LPS in the case of THP-1 cells.

In addition, we conducted Western blot analyses (Figure 4A and 4B) to further confirm the regulation of IL1a levels by miR-181a in Raw264.7 and THP-1 cell lysis solution as revealed by our ELISA data. We found that miR-181a significantly inhibited the increase in IL1a induced by LPS. Addition of miR-181a inhibitors led to slight increase in IL1a expression. These results are consistent with those obtained by ELISA and confirm the regulation of IL1a by miR-181a.

### Effects of miR-181a Precursors on the Levels of IL1a, IL1b, IL6, and TNFa

In this study, we found that transient transfection with pre-miR-181a precursors significantly decreased the levels of IL1a as well as IL1b, IL6, and TNFa in THP-1 cell lysis solution and culture medium (Figure 4C and 4D). However, anti-miR-181a precursors slightly increased the levels of IL1a, IL1b and IL6. These results are almost consistent with those obtained by using mature miR-181a mimics. However, miRNA precursors did not elicit stronger responses in these cells compared to miRNA mimics as we had expected. Changes in dosage and transfection method of miRNA precursors should be considered in future studies.

### Effects of Stable Expression of miR-181a in Raw264.7 Cells Exposed to LPS

**Figure 5:**
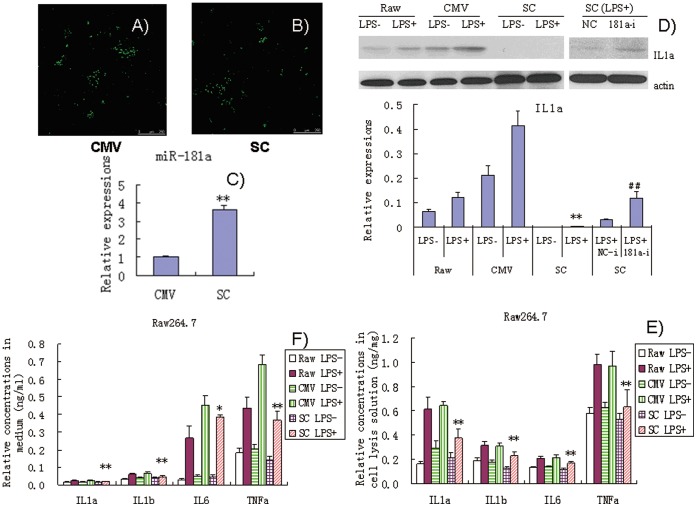
Effects of stable expression of miR-181a on production of inflammatory factors in LPS-induced Raw264.7 cells. (A) pCMV recombinant Raw264.7 cells (CMV, negative controls); (B) pCMV-pre-miR-181a recombinant Raw264.7 cells (SC); (C) endogenous miR-181a expression in SC (containing miR-181a expression vector) and CMV (containing negative control vector) cells; (D) Expression of IL1a in pCMV and pCMV-pre-miR-181a recombinant Raw264.7 cells assayed by Western blotting; (E and F) Levels of IL1a, IL1b, IL6, and TNFa in cell lysis solution and culture media of recombinant Raw264.7 cells transfected with pCMV and pCMV-pre-miR-181a. Data are expressed as mean ± SD (n = 3), *P<0.05, **P<0.01 vs. pCMV recombinant controls induced by LPS (CMV LPS+), ^##^ *P*<0.01 vs. miRNA inhibitor negative control (NC-i). LPS+ and LPS− indicate that cells were treated with and without LPS, respectively.

Transient transfection with exogenous miRNA mimics cannot imitate the physiological or pathological states of endogenous miRNAs as miRNA mimics are easily degraded. Stable expression of high levels of miRNA may be necessary to disclose their biological functions. We established a cell line with stable expression of high level of miR-181a via G418 selection in Raw264.7 cells (Figure 5A–5C). Cells transfected with the pCMV vector was used as NC. Results of western blot assays showed that stable expression of miR-181a significantly inhibited IL1a production (Figure 5D). ELISA results showed that stable expression of miR-181a significantly inhibited the production of IL1a as well as IL1b, IL6, and TNFa in Raw264.7 cell lysis solution and culture medium (Figure 5E–5F). Furthermore, miR-181a inhibitors significantly increased the production of IL1a in Raw264.7 cells with stable expression of miR-181a, as revealed by Western blot analysis (Figure 5D). This result further lends further support to the idea that miR-181a inhibits IL1a expression.

### Effects of Transient Transfection with miR-181a Vector in PMA/LPS-induced THP-1 Cells

**Figure 6:**
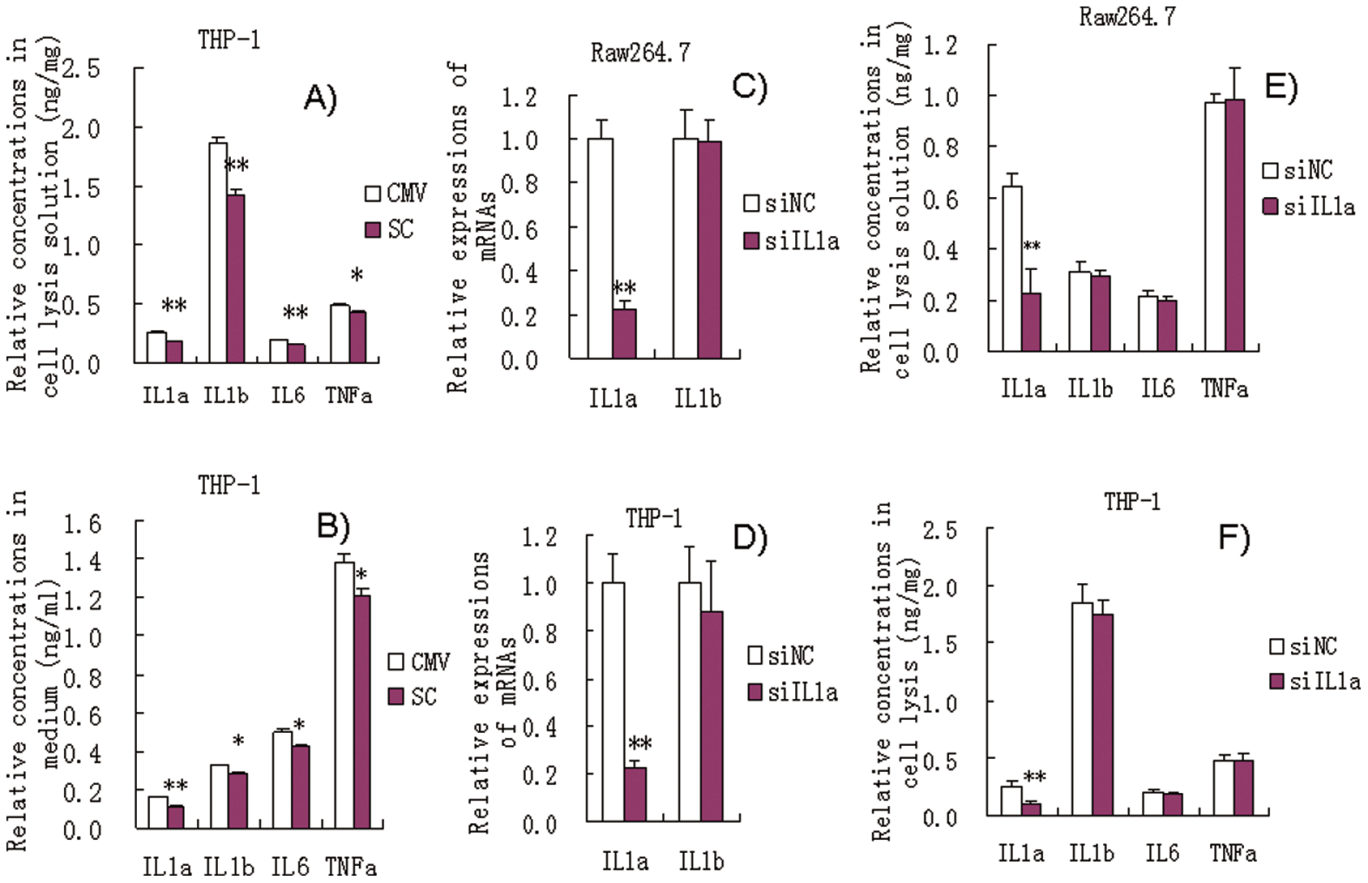
(A and B) Effects of transient transfection with miR-181a expression vector on the levels of ILa, IL1b, IL6, and TNFa in PMA/LPS-induced THP-1 cell lysis solution and culture media. Data are expressed as mean ± SD (n = 3), *P<0.05, **P<0.01 vs. CMV controls. ‘SC’, pCMV-pre-miR-181a recombinant THP-1 cells; ‘CMV’, pCMV recombinant THP-1 cells (negative controls). (C and D) Effects of si-IL1a on the mRNA levels of IL1a and IL1b in LPS-induced Raw264.7 and PMA/LPS-induced THP-1 cells. (E and F) Effects of si-IL1a on the levels of IL1a, IL1b, IL6, and TNFa in LPS-induced Raw264.7 and PMA/LPS-induced THP-1 cells. Data are expressed as mean ± SD (n = 3), *P<0.05, **P<0.01 vs. negative control (NC). ‘siNC’ is a single-stranded nucleic acid used as negative control for siIL1a.

Transient transfection with the pCMV-pre-miR-181a vector also significantly inhibited the production of IL1a, IL1b, IL6, and TNFa in the cell lysis solution and culture medium of PMA/LPS-induced THP-1 cells compared with those transfected with pCMV alone (Figure 6A and 6B). These results further support the idea that miR-181a has anti-inflammatory effects.

### Effects of si-IL1a on the Levels of IL1a, IL1b, IL6, and TNFa in LPS-induced Raw264.7 and PMA/LPS-induced THP-1 Cells

Next, we examined whether miR-181 has similar effects as siIL1a on the levels of IL1a, IL1b, IL6, and TNFa. siIL1a significantly inhibited IL1a mRNA expression by 80% in LPS-induced Raw264.7 and PMA/LPS-induced THP-1 cells, but no effects were observed on the level of IL1b mRNA (Figure 6C and 6D). Furthermore, siIL1a significantly down-regulated IL1a by 60% but had no significant effect on IL1b, IL6, and TNFa in cell lysis solution of both Raw264.7 and THP-1 (Figure 6E and 6F). These results indicate that siIL1a specifically knocks down IL1a. Thus, it appears that the bioactivities of miR-181a differ from those of siIL1a. However, further studies are needed to support this.

### Effect of miR-181a Mimics on the Levels of IL1a, IL1b, IL6, and TNFa in IL1a-induced THP-1 Cells

**Figure 7:**
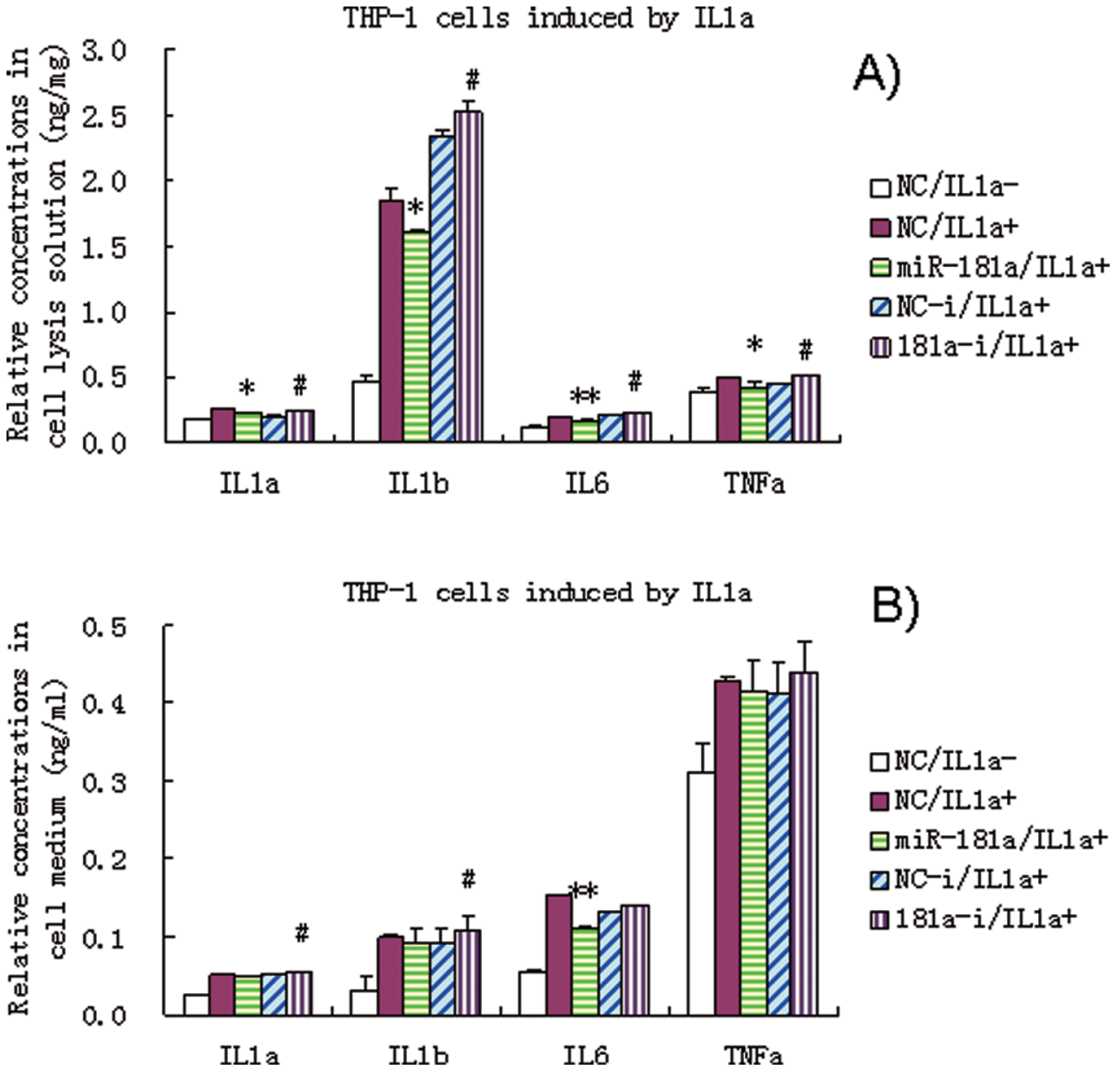
Effects of miR-181a mimics and inhibitors on the levels of IL1a, IL1b, IL6, and TNFa in IL1a-induced THP-1 cell lysis solution (A) and culture medium (B). Data are expressed as mean ± SD (n = 3), *P<0.05, **P<0.01 vs. negative control (NC), ^#^ *P*<0.05 vs. miRNA inhibitor negative control (NC-i). ‘NC-i’ is a single-stranded nucleic acid used as a negative control for miR-181a inhibitors (181a–i). IL1a + and IL1a- indicate that the cells were treated with (at a final concentration of 0.1 ng/ml) and without exogenous IL1a, respectively.

IL1a was exogenously applied to THP-1 cell media. IL1a induced significant increase in several inflammatory factors, namely, IL1b, IL6, TNFa, including endogenous IL1a. Importantly, miR-181a mimics still exhibited anti-inflammatory effects in IL1a-induced THP-1 cells (Figure 7A and 7B). These results further suggest that miR-181a exerts anti-inflammatory effects that involve other targets besides of IL1a.

### Effects of miR-181a Mimics on ROS in PMA/LPS Induced TPH-1 Cells

**Figure 8:**
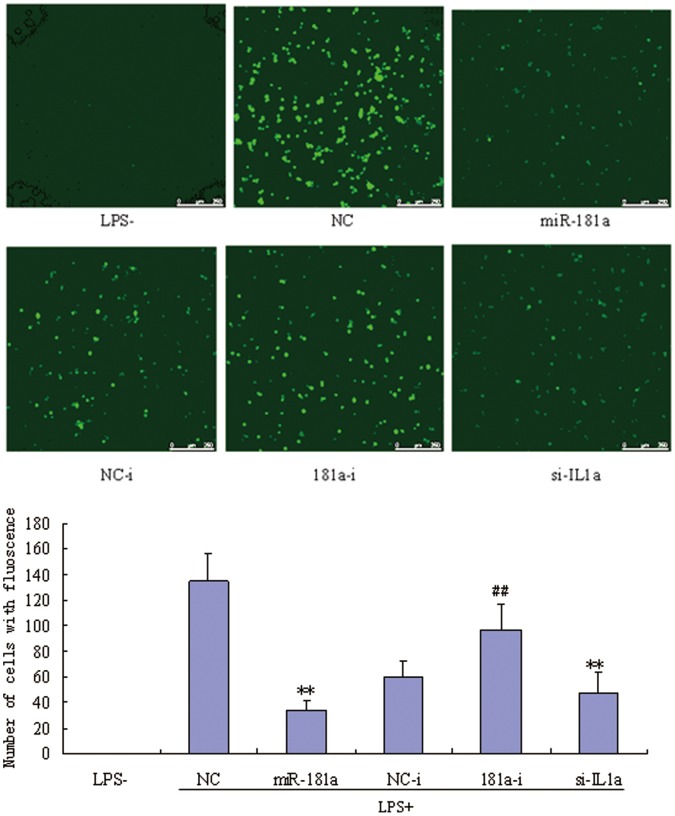
Effects of miR-181a mimics and inhibitors on the productions of reactive oxygen species (ROS) in PMA/LPS-induced TPH-1 cells. Data are expressed as mean ± SD (n = 3), **P<0.01 vs. negative control (NC), ^##^ *P*<0.01 vs. miRNA inhibitor negative control (NC-i). ‘NC-i’ is a single-stranded nucleic acid used as negative control for miR-181a inhibitors (181a–i). LPS+ and LPS− indicate cells treated with and without PMA/LPS, respectively.

Inflammation is associated with increase of ROS, which in turn, contributes to the development of inflammation. We found that miR-181a mimics significantly inhibited the production of ROS in PMA/LPS-induced THP-1 cells compared with NCs (Figure 8). However, miR-181a inhibitors slightly increased ROS levels, while siIL1a significantly decreased ROS levels compared to NCs. These results indicate that miR-181a likely attenuates the inflammatory state partly mediated by inhibiting IL1a.

## Discussion

Previous studies have reported that miR-181a regulates tumor growth and the immune system [16]. Although miR-181a has been reported to promote gastric cancer by negatively regulating the KLF6 tumor suppressor and the immune system [17], most studies indicate that miR-181a has anti-tumor effects. Overexpression of miR-181a effectively suppresses cell growth and induces G2-phase arrest and apoptosis partially by targeting RalA in leukemic K562 cells [18]. miR-181a has tumor suppressive effects against oral squamous cell carcinoma cells by downregulating K-ras [19]. miR-181a also functions as a tumor suppressor in human glioma cells [20]. miR-181a acts as an intrinsic antigen sensitivity “rheostat” during T cell development [21] and miR-181a inhibitors enhance mature T cell responses [22].

Aside from regulating tumor growth and the immune system, other biological functions of miR-181a have also been identified. miR-181a regulates vascular development and neo-lymphangiogenesis by targeting Prox1 [23]. Down-regulation of miR-181a up-regulates sirtuin-1 and improves hepatic insulin sensitivity [24]. miR-181a also regulates drug-induced synaptic plasticity by targeting AMPA-type glutamate receptors [25]. miR-181a regulates GRP78 and influences the outcome from cerebral ischemia *in vitro* and *in vivo* [26]. Moreover, miR-181a provides feedback regulation to TNF-α-induced transcription of pro-inflammatory genes in liver epithelial cells by suppressing p300/CBP-associated factor [27]. miR-181a has been reported to regulate TLR/NFκB signaling in monocytes of obese patients, but this inference was based only on q-PCR results and prediction analysis [28]. Whether miR-181a has anti-inflammatory functions still remains elusive. This study provides the first evidence of anti-inflammatory effects of miR-181a mediated by targeting IL1a in monocytes and macrophages. Our findings reveal a new biological function of miR-181a and also highlight its key pathophysiological implications.

IL1a is produced mainly by activated macrophages, neutrophils, epithelial cells, and endothelial cells. IL1a is one of the most important factors responsible for the production of systemic inflammation [29]. In this study, we investigated the regulatory role of a miRNA on Il1a in activated monocytes and macrophages since these cells play important roles in the development of systemic inflammation. We found the concentration of IL1a to be low in the cell culture media, but relatively high in the cell lysis solution. This result suggests that IL-la is not readily released from cells even upon activation, which is consistent with previous reports [30]. IL1a usually binds to IL1R, activates TNFa, and induces IL-6 secretion [31]. Aside from these functions, upregulation of inflammatory stimuli results in the translocation of IL1a to the nucleus and activation of the synthesis of proinflammatory cytokines [30].

In this study, LPS induced expression of miR-181a at low levels in THP-1 monocytes and Raw264.7 macrophages, while IL1a expression was increased. This indicates that miR-181a may play an important role in regulating the inflammatory state. Moreover, decreasing miR-181a levels by using miR-181a inhibitors would further deteriorate the inflammatory state. Previous studies reported that most miRNAs (e.g., miR-146a and miR-155) can play roles as both negative and positive modulators and are upregulated after inflammatory stimuli, which may be useful in maintaining cell homeostasis. Here, miR-181a likely provided positive feedback to inflammation stimulated by LPS in these specific cell lines. In a preliminary study, these phenomena were also seen in Hut102 and HepG2 cells (data not shown). Future studies should investigate whether miR-181a has similar roles in other cells as they do in monocytes or macrophages.

LPS binds to TLR4, activates NFKB and MAPK signaling pathways, and causes the expression and release of inflammatory factors such as IL1, IL6, and TNFa [32], [33]. IL1, IL6, and TNFa form a positive feedback loop to promote the inflammation process. These inflammatory factors are usually responsible for the systemic effects of inflammation. In this study, we found that exogenous application of IL1a alone was sufficient for the production of inflammatory factors, which is consistent with the findings of a previous report [34]. ROS are usually implicated in cellular inflammatory responses. PMA activates protein kinase C (PKC) and leads to ROS production [35]. ROS increase oxidative stress, activate the NFKB and MAPK signaling pathways, and promote the production of inflammatory factors such as IL1, IL6, and TNFa [36], which in turn increase ROS [37]. In addition, the binding of TLR4 to LPS further triggers ROS production [38]. Thus, PMA and LPS should have synergistic effects on the production of ROS and inflammatory factors. Chronic increase of ROS significantly damages cell structure. Excessive production of ROS associated with inflammation leads to oxidative stress, which is implicated in high mortality observed in several diseases, such as endotoxic shock [39].

**Figure 9:**
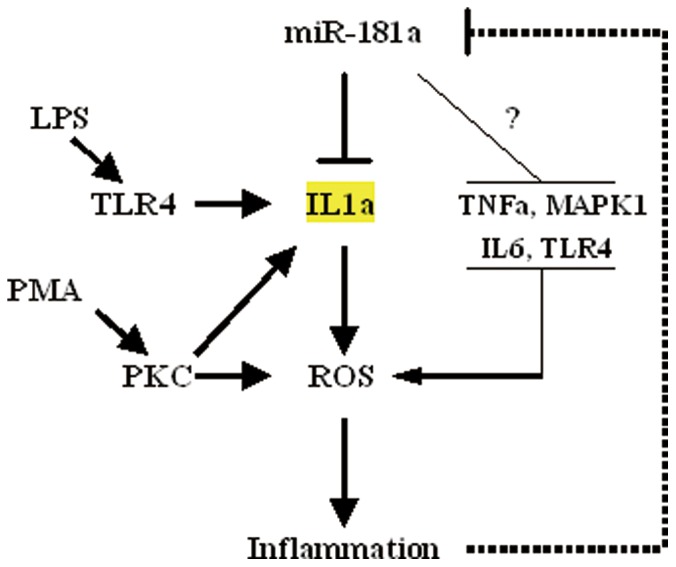
Potential effects and mechanisms of the regulation of inflammation by miR-181a.

In this study, THP-1 cells treated with PMA combined with LPS showed greater increase in ROS and inflammatory factors than LPS or PMA alone. This result was also observed in Raw264.7 cells (data not shown). Moreover, miR-181a inhibited the production of ROS, suggesting that miR-181a contributes to improvement in the inflammatory state. Silencing of IL1a was also associated with attenuation of ROS, suggesting that miR-181a improves inflammation at least in part by regulating IL1a levels. In this study, PMA was identified as a key factor for the production of ROS. PMA has been previously reported to activate inflammasomes and induce IL1a secretion [40]. The increase in IL1a may trigger production of ROS. Knock down of IL1a had no significant effects on the productions of IL1b, IL6, and TNFa, whereas miR-181a significantly inhibited IL1a, IL1b, IL6, and TNFa levels, suggesting that miR-181a functions differently compared to siIL1a. Considering previous results and predictions from bioinformatic analyses, it is possible that miR-181a exerts anti-inflammatory effects by involving other targets besides IL1a (Figure 9). Future studies should include experiments to examine these predictions.

### Conclusions

In summary, this study provides evidence for anti-inflammatory effects of miR-181a which is mediated at least in part by targeting IL1a in mice and human monocytes and macrophages. miR-181a regulates inflammatory factors through a positive feedback mechanism. These findings reveal a new biological function of miR-181a and highlight its pathophysiological implications in the field of inflammation and immunology.

## Supporting Information

Figure S1 Transfection with NC-Fam in Raw264.7 and THP-1 cells (A and B); miR-181a expression in Raw264.7 and THP-1 cells treated with miR-181a mimics and its inhibitors (C and D). Data are expressed as mean ± SD (n = 3), **P<0.01 vs. negative control (NC), ^##^ *P*<0.01 vs. miRNA inhibitor negative control (NC-i). ‘NC-i’ is a single-stranded nucleic acid used as negative control for miR-181a inhibitors (181a-i).
(TIF)
