# Supplementary figures and images for: miR-181a Regulates Inflammation Responses in Monocytes and Macrophages
Source: PLoS One. 2013 Mar 13;8(3):e58639. doi: 10.1371/journal.pone.0058639 (PMC3596280; doi:10.1371/journal.pone.0058639)

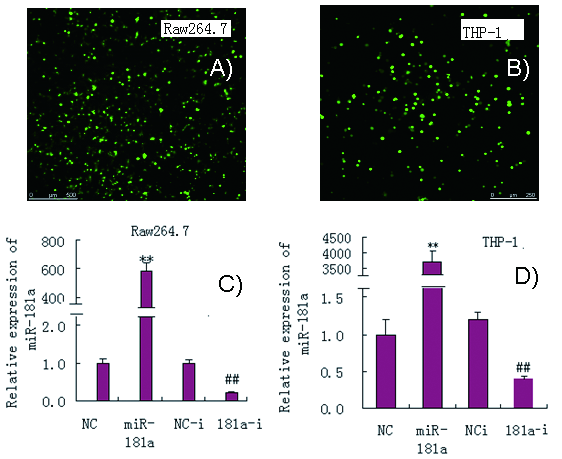

Supplement: Figure S1 — Transfection with NC-Fam in Raw264.7 and THP-1 cells (A and B); miR-181a expression in Raw264.7 and THP-1 cells treated with miR-181a mimics and its inhibitors (C and D). Data are expressed as mean ± SD (n = 3), **P<0.01 vs. negative control (NC), ## P<0.01 vs. miRNA inhibitor negative control (NC-i). ‘NC-i’ is a single-stranded nucleic acid used as negative control for miR-181a inhibitors (181a-i). (TIF) [file pone.0058639.s001.tif]
